# Supplementary material for: Pramipexole restores behavioral inhibition in highly impulsive rats through a paradoxical modulation of frontostriatal networks
Source: Transl Psychiatry. 2024 Feb 9;14:86. doi: 10.1038/s41398-024-02804-3 (PMC10858232; doi:10.1038/s41398-024-02804-3)
Supplement: Supplementary file 1 — Supplemental information [file 41398_2024_2804_MOESM1_ESM.docx]

# **Title**

**Pramipexole restores behavioral inhibition in highly impulsive rats through a paradoxical modulation of frontostriatal networks**

# **Authors**

Robin Magnard^1^, Maxime Fouyssac^2^, Yvan M. Vachez^1^, Yifeng Cheng^3^, Thibault Dufourd^1^, Carole Carcenac^1^, Sabrina Boulet^1^, Patricia H. Janak^3,4^, Marc Savasta^1^, David Belin^2^* and Sebastien Carnicella^1^*.

# **Affiliations**

^1^ Univ. Grenoble Alpes, Inserm, U1216, Grenoble Institut Neurosciences, 38000 Grenoble, France

^2^ Department of Psychology, University of Cambridge, Downing Street, CB2 3EB, Cambridge, United Kingdom

^3^ Department of Psychological and Brain Sciences, Johns Hopkins University, Baltimore, MD, 21218, USA

^4^ Solomon H. Snyder Department of Neuroscience, Johns Hopkins School of Medicine, Johns Hopkins University, Baltimore, MD 21205, USA

*Co-senior author

# **Correspondence:**

**Dr Robin Magnard; Present address:** Department of Psychological and Brain Sciences, Johns Hopkins University, Baltimore, MD, 21218, USA

***Phone: +*1(410)-516-7994**

***Email address:*** [**robin.magnard@jhu.edu**](mailto:sebastien.carnicella@inserm.fr)

**SUPPLEMENTAL FIGURE LEGENDS**

**Supplemental Figure 1: Regions of interest (ROIs) used for the quantification of immediate early genes expression.**

(**A**) The ROIs used for the quantification of the mRNA levels of the markers of cellular activity and plasticity C-fos and Zif268, respectively, in regions of the medial prefrontal cortex (mPFC) (**A**) and the striatum (**B**) are depicted in blue dashed line circles. (**A**) In the mPFC, from ventral to dorsal coordinates are represented the ROIs for Infralimbic (IL), Prelimbic (PrL), and Cingulate (Cg) cortexes. (**B**) In the striatum, from ventro-median to dorsolateral coordinates are represented the ROIs for Nucleus Accumbens Shell (NAc Shell), Nucleus Accumbens Core (Nac Core), Dorsomedial Striatum (DMS), and Dorsolateral Striatum (DLS). Background optical density, measured in the corpus callosum and as depicted here as grey dashed line circles, was subtracted to that of each ROI averaged across hemispheres.

## Supplemental Figure 2: Impulsivity expressed under treatment is not accounted for response rate before treatment.

(**A**) Full cohort, (**B**) LI rats, (**C**) MI rats, (**D**) HI rats, premature responding under treatment regressed by response rate during the last baseline (BL) session before treatment. (**E**) Full cohort, (**F**) LI rats, (**G**) MI rats, (**H**) HI rats, premature responding under treatment regressed by response rate during the last long intertrial interval (LITI) session before treatment. LI-Veh n= 5; LI-PPX n = 6; MI-Veh n = 10; MI-PPX n = 12; HI-Veh n = 6; HI-PPX n = 5. LI: low impulsive; MI: moderately impulsive, HI: highly impulsive.

## Supplemental Figure 3: PPX does not induce no perseverative behavior.

(**A**) Percentage of perseverative responses made across sessions. (**B**) Number of magazine head entries. LI-Veh n= 5; LI-PPX n = 6; MI-Veh n = 10; MI-PPX n = 12; HI-Veh n = 6; HI-PPX n = 5. BL: baseline; LITI: long intertrial interval; LI: low impulsive; MI: moderately impulsive; HI: higlhy impulsive. Data are shown as means ± SEM. LI-Veh vs. LI-PPX ^$^p<0.05. MI-Veh vs. MI-PPX ^££^p<0.01.

## Supplemental Figure 4: Zif268 and C-fos expression covariance amongst structures of the corticostriatal circuitry.

Spearman correlation matrix for C-fos and Zif268 in the seven structures stained for *in situ* hybridization. Color-code represents the $\text{ρ}$ value for each covariance, colder colors represent smaller $\text{ρ}$ coefficient. LI-Veh n= 5; LI-PPX n = 6; MI-Veh = 8; MI-PPX = 9; HI-Veh n = 6; HI-PPX n = 5. LI: low impulsive; HI: highly impulsive. IL: infralimbic cortex; PrL: prelimbic cortex; Cg: cingulate cortex; DLS: dorsolateral striatum; DMS: dorsomedial striatum; NAc Core: nucleus accumbens core; NAc Shell: nucleus accumbens shell. Significance covariance: *p<0.05; **p<0.01; ***p<0.001.
