# Supplementary figures and images for: Pramipexole restores behavioral inhibition in highly impulsive rats through a paradoxical modulation of frontostriatal networks
Source: Transl Psychiatry. 2024 Feb 9;14:86. doi: 10.1038/s41398-024-02804-3 (PMC10858232; doi:10.1038/s41398-024-02804-3)

A

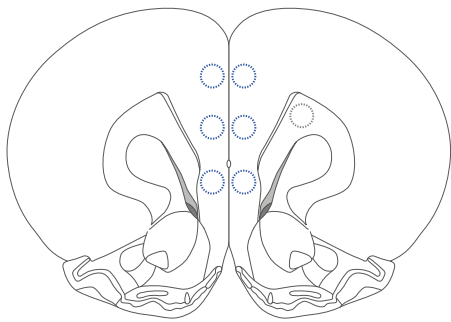

B

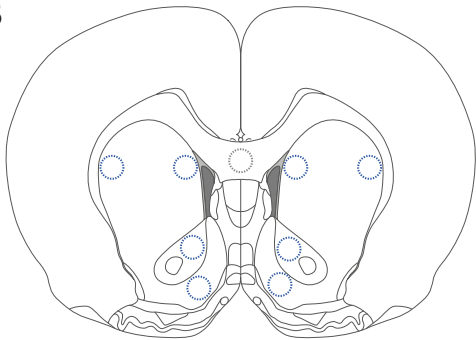

Supplement: Supplementary file 2 — Supplemental Figure 1 [file 41398_2024_2804_MOESM2_ESM.pdf]

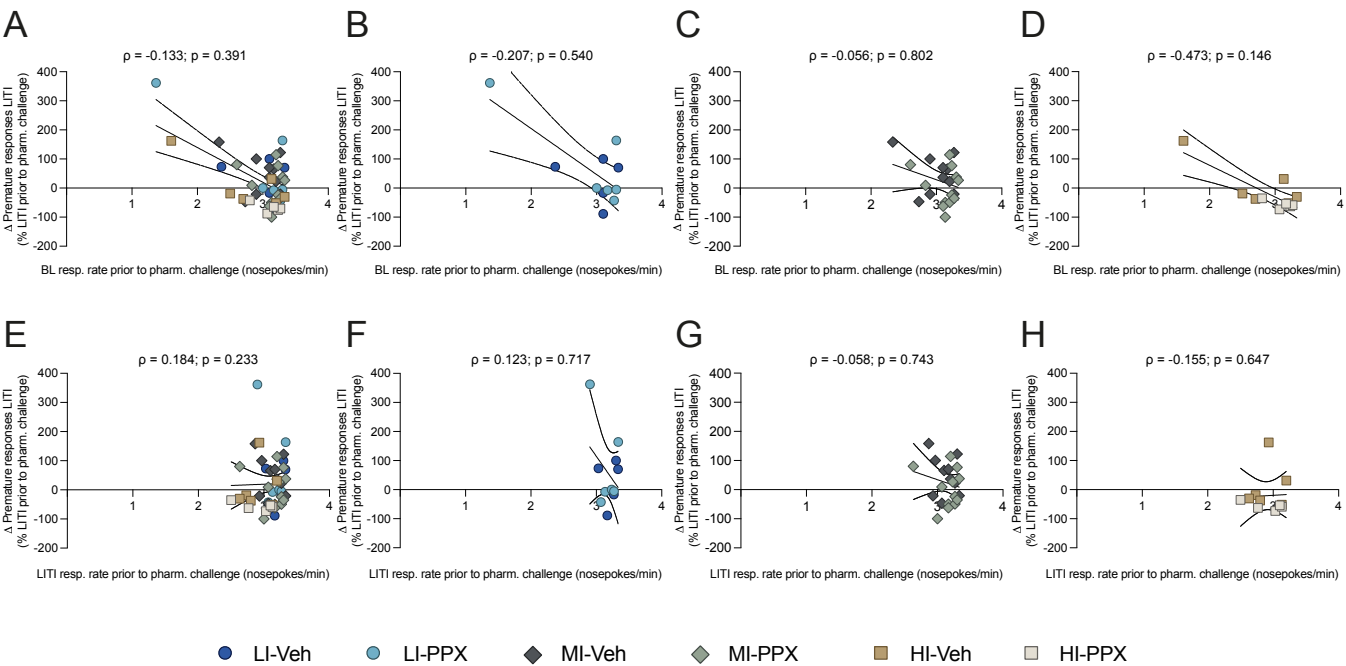

Supplement: Supplementary file 3 — Supplemental Figure 2 [file 41398_2024_2804_MOESM3_ESM.pdf]

A

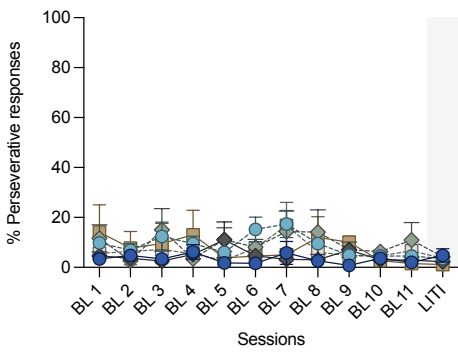

LI-Veh LI-PPX MI-Veh MI-PPX HI-Veh HI-PPX

B

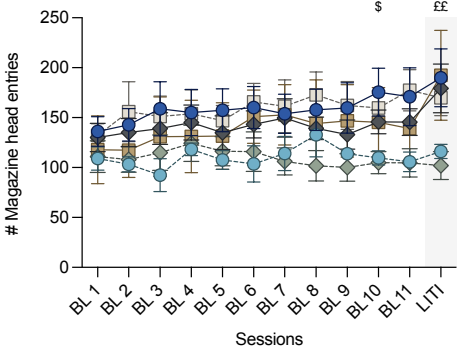

Supplement: Supplementary file 4 — Supplemental Figure 3 [file 41398_2024_2804_MOESM4_ESM.pdf]

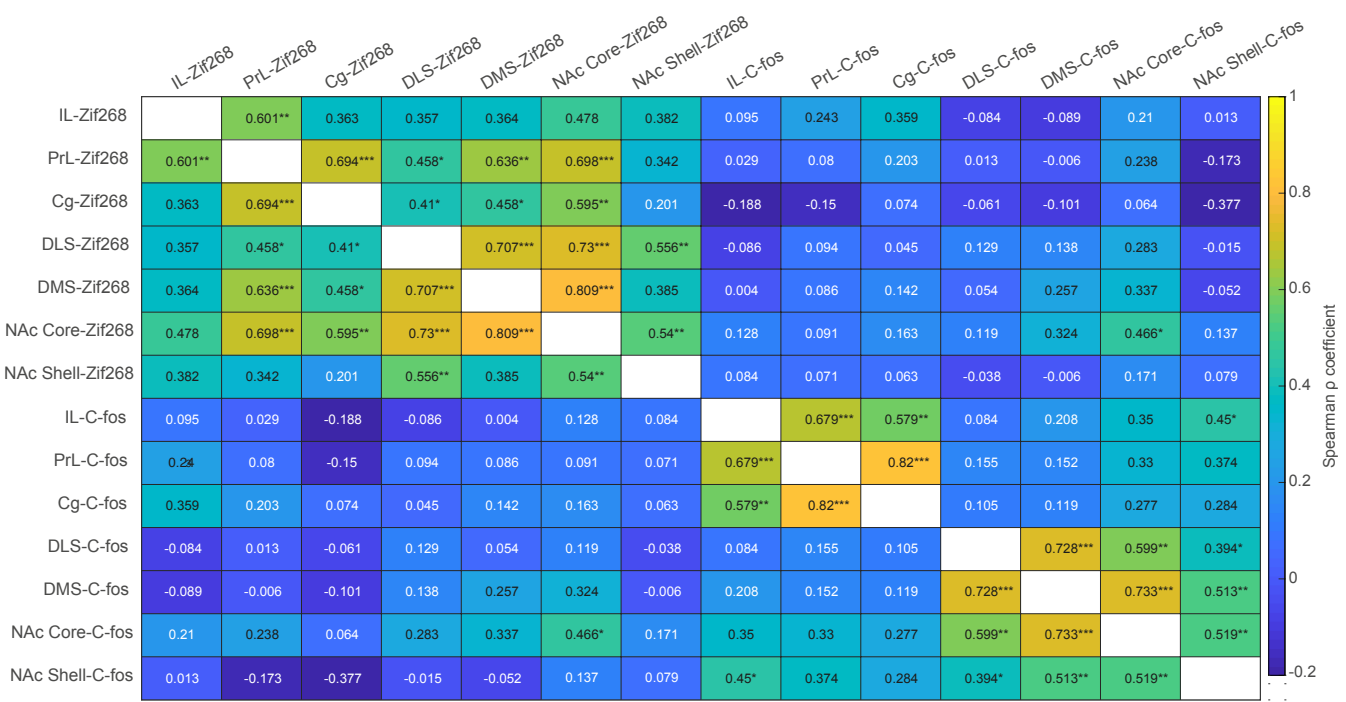

Supplement: Supplementary file 5 — Supplemental Figure 4 [file 41398_2024_2804_MOESM5_ESM.pdf]
